# Osteogenic Differentiation of Periodontal Ligament Stem Cells Seeded on Equine-Derived Xenograft in Osteogenic Growth Media

**DOI:** 10.3390/medicina58111518

**Published:** 2022-10-25

**Authors:** Montaser N. Alqutub, Areej H. Mukhtar, Yasser Alali, Fahim Vohra, Tariq Abduljabbar

**Affiliations:** 1 Department of Periodontics and Community Dentistry, College of Dentistry, King Saud University, Riyadh 11545, Saudi Arabia; 2 Department of Maxillofacial Surgery, College of Dentistry, King Saud University, Riyadh 11545, Saudi Arabia; 3 Prosthetic Dental Sciences Department, College of Dentistry, King Saud University, Riyadh 11545, Saudi Arabia

**Keywords:** PDLSC, xenograft, bone blocks, osteogenic differentiation, ELISA, collagen type I, calcium

## Abstract

*Background and Objectives*: The duration of bone turnover is critical, and different time points help in identifying the optimal endpoint of treatment duration. However, investigating the combination of xenograft and stem cells to allow tissue regeneration within an ideal time duration remains an under-investigated topic. The current study aimed to assess the impact of equine-derived xenograft bone blocks in assisting the human periodontal ligament stem cells (PDLSCs) to demonstrate osteogenic differentiation (collagen type 1 expression and calcium deposition) within an osteogenic growth media. *Materials and Methods*: Human PDLSCs were acquired commercially and seeded onto xenograft bone blocks. After the 14th and 21st day of culture, enzyme-linked immunoassay (ELISA) was utilized for the detection and quantification of levels of collagen type I, while the mineralization assessment (deposition of calcium) was conducted by staining the PDLSCs with Alizarin Red S (ARS). The statistical comparison between the means and standard deviations of study groups were evaluated using analysis of variance (ANOVA). *Results*: ELISA assessment revealed an upsurge in the expression of collagen type I for PDLSCs cultured with xenograft after 14 and 21 days compared to the controls (intergroup comparisons significant at *p* < 0.05). Similar findings were obtained for mineralization assessment and on ARS staining. PDLSCs cultured with xenograft bone blocks presented an increased deposition of calcium compared to their control counterparts (intergroup comparisons significant at *p* < 0.05). *Conclusions*: PDLSCs embedded in xenograft bone blocks inside an osteogenic growth medium demonstrated greater osteogenic differentiation potential after 14 and 21 days. This superior osteogenic differentiation capability was evident by increased collagen type I expression and more significant calcium deposition at the 14th and 21st days after culture.

## 1. Introduction

Bone tissue engineering’s success is dependent on the synergistic interaction between the progenitor cells and the grafts [1]. For periodontal tissue regeneration (including the alveolar bone), stem cells from various sources can be combined with bone grafts [2,3]. Stem cells are generally characterized as embryonic stem cells (ESCs), induced pluripotent stem cells (iPSCs), and mesenchymal stem cells (MSCs) [4]. The MSCs have a pronounced potential in regenerative medicine as they are multipotent, self-renewable, and culturally expandable in vitro with outstanding genomic permanency [5]. Concerning dentistry, within the MSCs, periodontal ligament stem cells (PDLSCs) are essential as they have been shown to stimulate periodontal regeneration [6]. The PDLSCs are considered somatic cells that can distinguish into different cell types and go through vigorous clonal self-renewal [7]. In addition, they express runt-related transcription factor 2 (RUNX-2) and alkaline phosphatase (ALP), which are related to the conversion of MSCs into osteoblasts and promoting bone mineralization, respectively [8]. Therefore, PDLSCs are an extremely promising stem cell group for regeneration, particularly related to the human periodontium.

In addition to progenitor cells, tissue engineering mediations depend on bone grafts such as autografts, allografts, and xenografts [9]. Autografts are a gold standard due to their remarkable osteogenic properties but also retain some shortcomings that include donor site morbidity and restricted availability [10]. Allografts were introduced to overcome the problems linked with autografts. Allografts are bone grafts obtained from similar species and hold osteoconductive potential [11]. Nevertheless, allografts are associated with immune rejection and disease progression [12]. Xenografts are bone substitutes obtained from non-human species and could be coral, bovine, or equine in origin [13]. Although xenografts also entail the risk of getting rejected by the recipient’s immune system, they are still being investigated for various clinical applications as human tissues (autografts) cannot be obtained easily in sufficient quantities [14]. A xenograft, which has lately gained admiration, is the equine-derived bone graft. The equine-derived xenograft has displayed substantial osteoinductive properties with the retention of various growth factors [15]. Previously, Di Stefano et al. confirmed that the equine bone could be effectively used for mandibular ridge augmentation in patients with horizontal mandibular ridge deficiencies [16]. In another study aimed at lateral augmentation of rat mandible, it was reported that equine bone blocks were able to form new bone that was significantly higher than bovine bone blocks [17]. Pistilli et al. also demonstrated similar findings and reported that equine bone blocks could be used to treat bone atrophy of the maxilla, and their use resulted in the growth of the width of edentulous alveolar crests [18]. Hence, equine-derived xenografts are suitable alternatives to autografts or allografts and have great potential in bone tissue engineering.

Duration of new bone formation (bone turnover) is a critical parameter [19], and different time points help in identifying the optimal endpoint of treatment duration. The investigation of the best combination of xenograft and stem cells to allow tissue regeneration within an ideal duration remains a valid question for the successful planning of bone regeneration procedures. Therefore, the present study aimed to appraise the impact of equine-derived xenograft bone blocks in assisting PDLSCs in demonstrating osteogenic differentiation (collagen type 1 expression and calcium deposition) inside an osteogenic growth media. It is hypothesized that PDLSCs cultured with xenogenic bone blocks would express more collagen type 1 and demonstrate an increased calcium deposition (osteogenic differentiation).

## 2. Materials and Methods

### 2.1. Ethical Approval

The current study was accomplished following the principles of the Helsinki Declaration of 1964 and its later amendments. The ethical approval was attained from the College of Dentistry’s Research Center (CDRC), King Saud University, and the Institutional Review Board.

### 2.2. PDLSCs Culture and Study Groups

Human PDLSCs were obtained commercially (ScienCell, Carlsbad, CA, USA). These cells were isolated from the human periodontal tissue, cryopreserved at −80 °C, and then delivered frozen in a vial containing >5 × 10^5^ cells in 1 mL volume. PDLSCs were cultured at passage-4 in a regular growth media and placed in a T-75 flask. The PDLSCs were permitted to mature, and the media was replenished every 24 h until confluency of 85% was obtained within two weeks post-culture. Using a hemocytometer, the cell counting was accomplished to warrant that cell proliferation and growth had touched the required confluency. The PDLSCs were seeded with 250,000 cells on 5 mm × 5 mm × 5 mm xenograft bone blocks, OsteoBiol SP-block Norm (Tecnoss Dental, Coazze TO, Italy). Based on the different combinations of xenograft, growth media, and time duration, the study comprised of four groups: Control-14: PDLSCs were cultured in an osteogenic growth media (prepared of basal media DMEM along with fetal bovine serum, beta glycerophosphate, dexamethasone, Vitamin C, Vitamin D, and penicillin streptomycin) only and assessed 14-days post-culture; Control-21: PDLSCs were cultured in an osteogenic growth media only and assessed 21-days post-culture; PDLSC-X-14: PDLSCs were cultured with a xenograft bone block in an osteogenic growth media and evaluated 14-days post-culture; and PDLSC-X-21: PDLSCs were cultured with a xenograft bone block in an osteogenic growth media and assessed 21-days post-culture. Each group contained six bone blocks cultured with PDLSCs in osteogenic growth media. For osteogenic induction, cells were retained inside regular growth media for 24 h, followed by replacement with osteogenic media. Using a 48-well plate, all the samples were then cultured with one block in each well and supplemented by 500 µL of the allocated culture media. This culture media was replenished every other day till the day of analysis.

### 2.3. Enzyme-Linked Immunoassay (ELISA) Assessment

Specific enzyme-linked immunoassay (ELISA) was utilized to detect and quantify levels of collagen type I, a bone-related protein. The ELISA kit used in this experiment included an antibody-coated 96-well plate (Molequle-ON (ELI-M-015-96) human collagen I Alpha). Media from all cultured wells was discarded, and all wells were washed with 1 mL of ice-cold phosphate buffer saline (PBS). The PBS was then discarded, and 1 mL of ice-cold lysis buffer (RIPA buffer) was supplemented to each well and agitated for 30 min at 4 °C. The cells were scraped and transferred along with the buffer into microfuge tubes, and the tubes were centrifuged at 16,000× *g* for 20 min to gather the supernatant. The extracted protein samples were stained with loading buffer solution (4X Protein Sample Loading Buffer for Western Blots, LI-COR, Lincoln, NE, USA), 100 µL was pipetted for each sample, and then incubated for 90 min at 37 °C. Upon the completion of the incubation period, the samples went through a cycle of washing (carried out by using 350 µL of wash buffer). Then, 100 µL of working solution (biotin-conjugated anti-human collagen I) was transferred to each well and incubated at 37 °C for 60 min. Post washing, 100 µL working solution (streptavidin-HRP) was added to each well and incubated at 37 °C for 30 min, followed by washing. 50 µL of stop solution was transferred to each well with gentle tapping of the plate to warrant uniform mixing, and the sample color change from blue to yellow was observed. Finally, the plate was placed in the microplate reader (Synergy™ HT Multi-Detection Microplate Reader, BioTek, Santa Clara, CA, USA) and read at 450 nm.

### 2.4. Mineralization Assessment

For the assessment of mineralization in the PDLSCs cultures, staining with Alizarin Red S (ARS) was used. The samples were fixed, and the fixation solution was added (200–300 µL of 4% paraformaldehyde in PBS was added in each well), and then the samples were kept at room temperature for 15 min. The fixation solution was discarded, and all samples were washed with distilled water (DW) twice. Subsequently, 1 mL of 2% ARS staining solution was pipetted to each well, the plates were concealed with aluminum foil and then incubated for 90 min at room temperature. These plates were gently swirled every 20 min to ensure uniform mixing. Following the incubation period, the stain was discarded from each well, and all the wells were washed with DW ten times. The samples were visualized by phase microscopy utilizing an inverted light microscope (Nikon, Tokyo, Japan), and the microscope images were acquired.

For the quantification of the stained calcium deposits, 800 µL of acetic acid was pipetted to each well, and the plates were incubated for 30 min at room temperature with mild shaking. The cells were acquired by scraping the plates and scaffolds and transferring the acetic acid to a 1.5 mL microcentrifuge tube. The tubes were vortexed for 30 s and 500 µL of mineral oil was pipetted to each tube. Afterward, all the tubes were heated gradually for 10 min to 85 °C and immediately moved to the ice for 5 min. The tubes were centrifuged at 20,000× *g* for 15 min, and 500 µL of the supernatant was removed and transferred to another 1.5 mL microcentrifuge tube. Then, 200 µL of 10% ammonium hydroxide (NH-OH) was supplemented, and the plates were read in the microplate reader (Synergy™ HT Multi-Detection Microplate Reader, BioTek) at 450 nm in 96-well plate format.

### 2.5. Statistical Analysis

The means and standard deviations for collagen type I and calcium deposition outcomes were compared statistically using statistical program for social sciences (SPSS, version 20, IBM Corporation, Somers, NY, USA). The statistical comparison between the means and standard deviations of study groups were evaluated using analysis of variance (ANOVA).

## 3. Results

### 3.1. Outcomes of the ELISA Assessment

**Figure 1:**
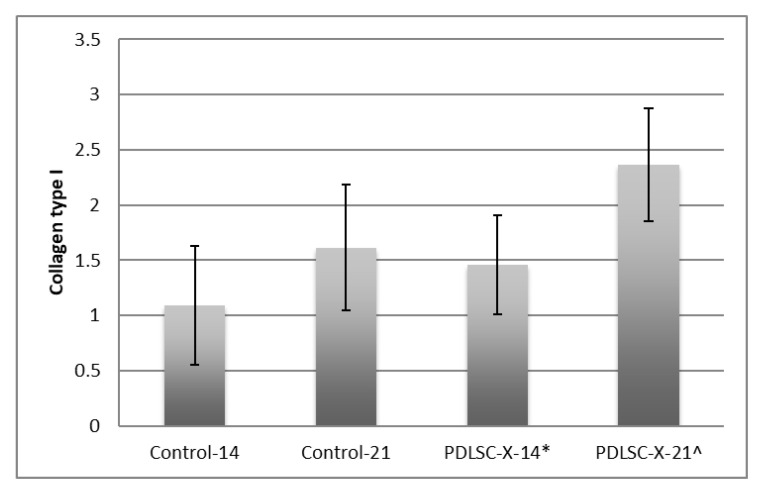
Mean collagen type I (μg) distribution according to the duration of culture and graft material among the study groups. * significant compared to Control-14, significant compared to Control-21.

ELISA assessment revealed a surge in the expression of collagen type I for PDLSC-X-14, and PDLSC-X-21 groups, compared to the Control-14 and Control-21 groups (Figure 1). On the 14th day, it was noticed that for the PDLSC-X-14 group, the collagen type I expression was 1.461 ± 0.45, while for the Control-14 group, it was 1.093 ± 0.54. On the 21st day, the collagen type I expression observed for the PDLSC-X-21 group was 2.367 ± 0.51, and for the Control-21 group, it was 1.613 ± 0.57. All the intergroup differences were statistically significant (*p* < 0.05).

### 3.2. Outcomes of the Mineralization Assessment

**Figure 2:**
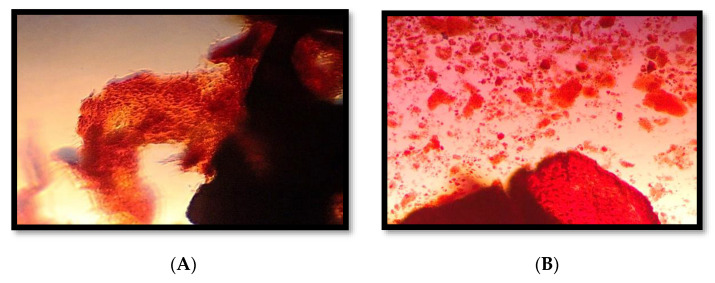
Alizarin Red S staining of mineralized calcium deposition. (**A**) 14 days of cultured PDLSCs with xenograft bone block in osteogenic growth media, (**B**) 21 days of cultured PDLSCs with xenograft bone block in osteogenic growth media.

**Figure 3:**
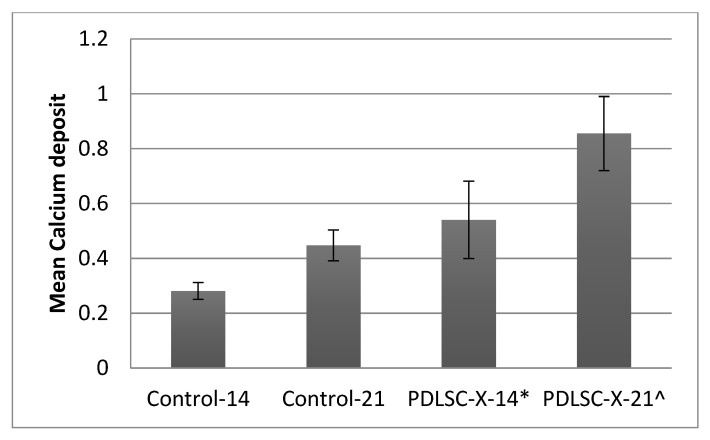
Mean calcium deposition among study groups (μg). * significant compared to Control-14, significant compared to Control-21.

On ARS staining, PDLSC-X-14 and PDLSC-X-21 groups revealed an increased calcium deposition compared to their control counterparts (Figure 2). On the 14th day, it was noticed that for the PDLSC-X-14 group, the calcium deposition was 0.54 ± 0.141, while for the Control-14 group, it was 0.281 ± 0.031. On the 21st day, the calcium levels observed for the PDLSC-X-21 group were 0.855 ± 0.135, and for the Control-21 group, they were 0.447 ± 0.056. All the intergroup differences were statistically significant (*p* < 0.05) (Figure 3).

## 4. Discussion

Based on the findings of this study, the hypothesis was accepted that the PDLSCs cultured with equine-derived xenogenic bone blocks demonstrated an increased expression of collagen type 1 and greater calcium deposition. Our study evaluated the osteogenic differentiation on the 14th and 21st day by determining the expression levels of collagen type 1 (using ELISA test) and by assessing the calcium deposition (using ARS staining).

In the present study, the ELISA assessment results demonstrated a rise in the expression of collagen type I for the PDLCs cultured on xenogenic bone blocks at both time points, compared with the controls. To achieve appropriate osteogenic differentiation levels, the cells should produce bone extracellular matrix (ECM) that functions as a scaffold upon which minerals are deposited [20]. A key component of ECM is collagen type I, which is responsible for binding all the deposited minerals [21]. Hence, an early expression of collagen type I is considered a positive indicator for osteogenic differentiation. Our results demonstrated the expression of collagen type I at the early stages of the differentiation on the 14th day, with an increased expression pattern observed on the 21st day. In a similar previous study, an increased expression of collagen type I was seen on the 21st day from MSCs isolated from adipose tissue [22]. This pattern of expression coincides with the developmental phases of osteogenesis and confirms that the early manifestation of collagen type I helps in producing an intact matrix for mineral deposition [23]. Therefore, the ELISA findings of our study demonstrate the outstanding osteogenic potential of all cultured cells (PDLSCs cultured with xenograft bone block in an osteogenic media performed better than the controls).

Concerning mineralization assessment (calcium deposition) results, again, PDLSCs cultured with xenograft bone blocks demonstrated an increased calcium deposition compared to the controls on 14th and 21st day. Since mineralization is considered a functional endpoint in in-vitro research that indicates advanced osteogenic cell differentiation, calcium deposition levels were detected using ARS staining. The rationale behind using this histological staining for this purpose is its capacity to specifically stain matrix containing calcium (indicating positive expression of bone-matrix deposition) [24]. Our findings showed more calcium deposition on the 21st day compared with the 14th day for both groups. In another earlier study, the osteogenic potential of PDLSCs was assessed using confocal microscopy and scanning electron microscopy (SEM) [25]. In the same study, it was reported that increased osteogenic differentiation was observed on the 21st day compared to the 14th-day post-culture. This could be elucidated by understanding the confluent osteogenic differentiation process, which goes through certain stages. In the first stage (between 1–4 days of culture), the cells begin to expand and proliferate, and in the second stage (between 5–14 days of culture), early cell differentiation starts, which is characterized by the expression of ALP and collagen type I [26]. The final stage (spanning between 14–28 days of culture) represents a high expression of osteocalcin and osteopontin, trailed by the deposition of calcium and phosphate minerals [26].

In the present study, we used PDLSCs and also investigated the influence of culture duration (14 and 21 days) on the osteogenic differentiation (collagen type 1 expression and calcium deposition) on these cells when they are cultured with xenogenic equine bone blocks in an osteogenic growth media. Although stem cells could be obtained from numerous tissues in the body, various studies have shown that dental-derived stem cells have unique benefits over other stem cells, including ease of collection, abundance in number, and the capability to differentiate into multiple other cell types [27,28,29]. Additionally, many studies have also verified that these dental-derived stem cells have superior osteogenic differentiation potential when matched with bone marrow-derived MSCs [30,31]. In light of these arguments, we decided to use PDLSCs in this study and analyze the impact of using xenograft bone blocks on their osteogenic differentiation potential.

Xenografts were used in the current study, and PDLSCs were cultured with them in osteogenic media. Previously, Mukhtar and Alqutub followed a similar methodology and reported in their study that equine-derived xenograft bone blocks are suitable to seed human PDLSCs and to confirm osteogenic capabilities [31]. Our study conforms with their findings, and we found that the osteogenic differentiation of PDLSCs cultured with equine-derived xenograft bone blocks was superior to the controls. Another study demonstrated that equine-derived xenograft provides optimal cell adhesion to the dental-derived stem cells and promotes the whole osteogenic process [32]. Our findings are again in agreement with this study, and we also found equine-derived xenograft bone blocks to facilitate the osteogenic differentiation process.

Our study had certain limitations, and the reader should cautiously interpret our findings. First, the present study was conducted in an in-vitro setting. The same experiments, if conducted in-vivo, could yield different results as the in-vivo environment is different, and it could offer multiple challenges to the xenografts or PDLSCs. These challenges could include the influence of saliva and the pressures exerted by mastication and occlusion. Moreover, our study was only conducted with xenograft bone blocks. Further studies using different types of bone grafts should be undertaken to investigate the true osteogenic differentiation potential of PDLSCs. Additionally, the PDLSCs were acquired from one commercial organization. It is possible that PDLSCs from another supplier, when used for the same experiments, could yield different results (due to different extraction and storage procedures).

## 5. Conclusions

Within the limitations of the study, PDLSCs embedded in xenograft bone blocks inside an osteogenic growth medium demonstrated improved osteogenic differentiation potential. This superior osteogenic differentiation capability was evident by increased collagen type I expression and greater calcium deposition on the 14th and 21st day after culture.

## Data Availability

The data are available on contact with the corresponding author.
